# Single‐step equipment‐free extracellular vesicle concentration using super absorbent polymer beads

**DOI:** 10.1002/jev2.12074

**Published:** 2021-02-23

**Authors:** Hee Cheol Yang, Yoo Min Ham, Jeong Ah Kim, Won Jong Rhee

**Affiliations:** ^1^ Department of Bioengineering and Nano‐Bioengineering Incheon National University Incheon Republic of Korea; ^2^ Center for Scientific Instrumentation Korea Basic Science Institute Chungbuk Republic of Korea; ^3^ Department of Bio‐Analytical Science University of Science and Technology Daejeon Republic of Korea; ^4^ Division of Bioengineering Incheon National University Incheon Republic of Korea

**Keywords:** exosome, extracellular vesicle concentration, extracellular vesicles, isolation, super absorbent polymer

## Abstract

Extracellular vesicles (EVs) contain useful biomarkers for disease diagnosis and are promising biomaterials for the delivery of therapeutic molecules *in vivo*. Accordingly, an efficient concentration method is necessary for large‐scale production or high‐throughput isolation of EVs from bulk liquid samples, including culture medium and body fluids, to achieve their clinical application. However, current EV concentration methods, including ultrafiltration, are limited with respect to cost, efficiency, and centrifugation time. In this study, we developed the first single‐step, equipment‐free EV concentration method using super absorbent polymer (SAP) beads. SAP beads absorb small molecules, including water, via nano‐sized channels but expel and thereby concentrate EVs. Consequently, the beads drastically enrich EVs by reducing the solution volume in a single step, without affecting EV characteristics. Moreover, the purity of the concentrated EV solution was high due to the absorption of protein impurities by SAP beads. To further demonstrate the versatility of the method, we showed that SAP beads successfully enrich EVs in human urine samples and culture medium, enabling better isolation performance than conventional ultrafiltration. We believe the newly developed approach and insight gained in this study will facilitate the use of EVs as prominent biomaterials for disease diagnosis and therapy.

## 1 INTRODUCTION

Extracellular vesicles (EVs) are small particles with sizes ranging from 50–1000 nm that are constantly secreted by cells (Raposo & Stoorvogel, 2013). Since EVs originate from cells, they contain cellular components such as proteins, peptides, lipids, carbohydrates, DNAs and RNAs (Merino et al., 2014; Schorey & Bhatnagar, 2008). As central mediators of intercellular communication, EVs, including exosomes and microvesicles, play important roles in the pathogenesis of various diseases, such as cancer progression and metastasis (Becker et al., 2016; Penfornis et al., 2016). The contents of EVs can be transferred to other cells in the same or a different tissue, thereby mediating cell‐to‐cell communication (Patil & Rhee, 2019). EVs enter the circulatory system and are therefore found in most body fluids, including the blood (Caby et al., 2005), urine, saliva (Ogawa et al., 2011) and breast milk (Admyre et al., 2007). As such, EVs are promising novel biomarkers for liquid biopsy to overcome the limitations of previously established biomarkers (Akers et al., 2013; Contreras‐Naranjo et al., 2017; Gamez‐Valero et al., 2015; Kinoshita et al., 2017; Nawaz et al., 2014). Recent studies have demonstrated the therapeutic potential of EVs (El Andaloussi et al., 2013; Samanta et al., 2016). For instance, nano‐sized EVs can successfully deliver drugs such as proteins, peptides, chemicals, and nucleic acids (Jeong et al., 2019, 2020).

Widespread use of EVs for diagnostic or therapeutic applications requires an efficient isolation process and the ability to purify EVs from various mixtures, including culture medium and human body fluids (Cho et al., 2020; Shtam et al., 2018). Over the past decade, remarkable progress has been made in the development of EV isolation technologies, including polymer‐based precipitation (Rider et al., 2016), ultracentrifugation (Momen‐Heravi, 2017), and size exclusion chromatography (SEC) (Böing et al., 2014). However, it is necessary to establish a process for EV concentration in a solution. For instance, a large volume of cell culture medium is needed to obtain a sufficient number of EVs from a scaled‐up bioreactor. Ultracentrifuge equipment usually process limited sample volumes. Moreover, the sample injection volume is limited in SEC and even the eluted fractions containing EVs purified by SEC are usually diluted, requiring additional concentration processes to obtain an EV concentration suitable for diagnostic or therapeutic purposes. Ultrafiltration is the most widely used EV concentration method (Vergauwen et al., 2017). However, this process is limited by EV trapping and membrane clogging (Liga et al., 2015), leading to a drastic reduction in yield after concentration and a short membrane lifetime. Moreover, the method is costly and requires equipment, such as centrifuges. Thus, the development of an efficient, low‐cost, high‐yield and fast method for EV concentration is needed.

Super absorbent polymers (SAPs) are hydrogels with the ability to absorb water weighing several hundred to thousand times their dry weight (Mudiyanselage & Neckers, 2008; Ramazani‐Harandi et al., 2006). It was reported that the size of water channel is 5 nm (Xie et al., 2016). SAPs absorb water through osmosis, which is driven by counter ions attached to the polymer as well as the physical water entrapment via capillary forces (Behera & Mahanwar, 2019; Wu et al., 2020; Zhang et al., 2014; Zohourian Mehr & Kabiri, 2008). Owing to the water‐absorbing properties, SAPs are a major component of diapers and are widely applied as hygienic products in the agriculture and food industry for protection and storage purposes (Ahmed, 2015; Chen et al., 2004). SAPs have been used as an alternative to filtration methods for concentrating microorganisms from water samples (Xie et al., 2016). However, studies focused on the bio‐industrial and biomedical applications of SAP are limited.

**FIGURE 1:**
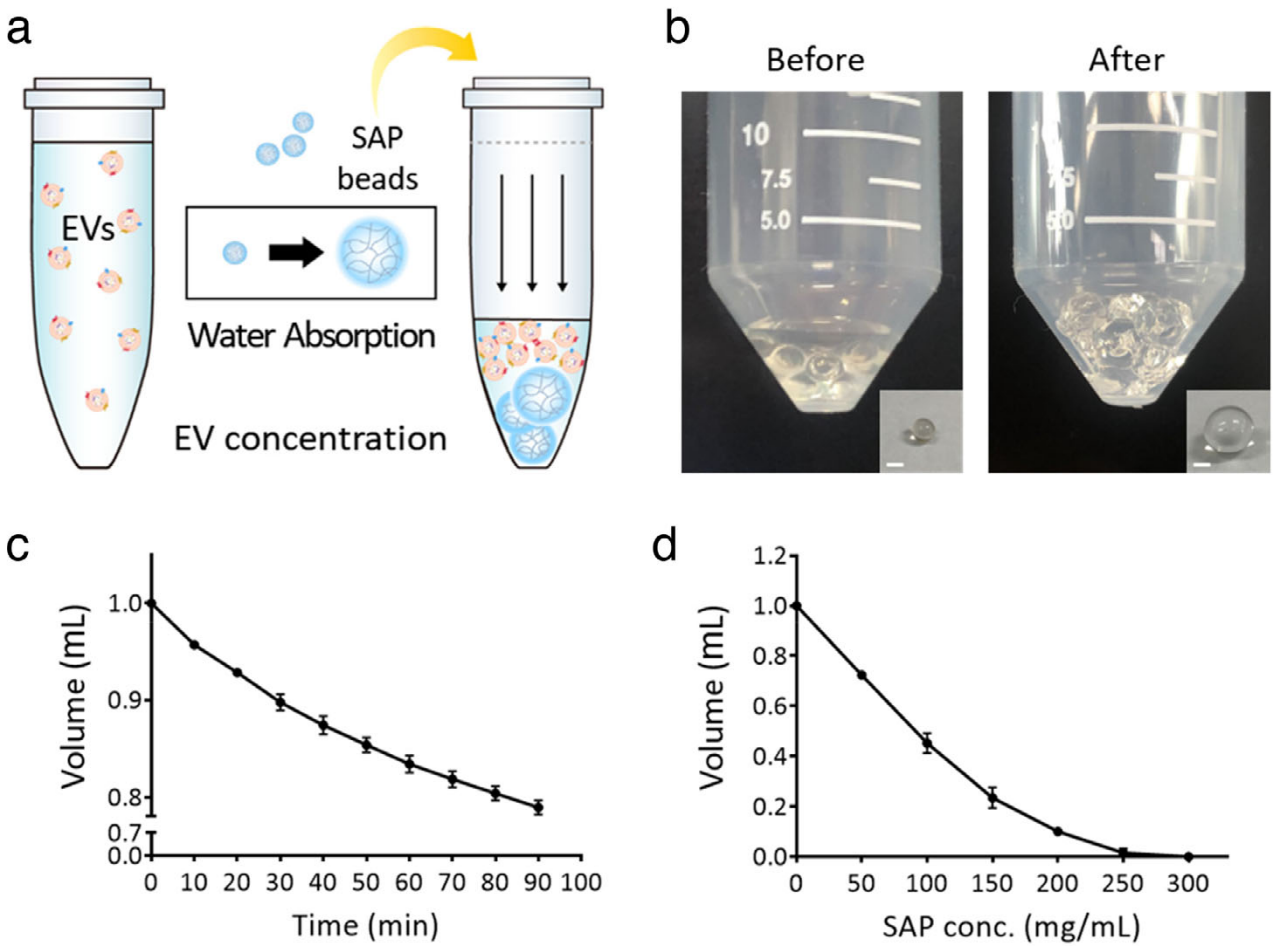
SAP bead‐based EV concentration method. [(a) Conceptual image of EV enrichment using SAP beads. (b) Swelling of SAP beads and absorption of PBS. SAP beads were incubated with the PBS solution. The inner boxes show the increased size of single SAP beads after incubation. Scale bar indicates 2 mm. (c) Time‐dependent water absorption and volume reduction profiles of single SAP beads. (d) Concentration‐dependent water absorption and volume reduction profiles of SAP beads for 30 min]

Herein, we developed the first single‐step method for EV concentration using SAP beads (Figure 1a). Based on the huge size difference between the water channel of SAP beads and EVs, we hypothesized that relatively small molecules including water would be absorbed by SAP beads; EVs would be excluded and thereby concentrated. The method is straightforward, effective, scalable and equipment‐free and can be widely applied in therapeutic or diagnostic applications requiring EV enrichment.

## 2 MATERIALS AND METHODS

### 2.1 Cell culture, EV‐free FBS preparation, and EV isolation from culture media

HeLa cells were cultured at a humidified atmosphere of 5% CO_2_ and 37°C in Dulbecco's Modification of Eagle's Medium (DMEM; Cellgro, USA) supplemented with 10% (v/v) fetal bovine serum (FBS; Gibco, USA) and 1% (v/v) penicillin‐streptomycin. For the production of EVs, HeLa cells were grown in a medium containing EV depleted FBS, generated by the centrifugation of FBS at 120,000 × *g* for 10 h at 4°C using a TLA‐100.3 fixed‐angle rotor with clearing factor (k factor) 44 in an ultracentrifuge (Optima TL‐100; Beckman Coulter, USA). The supernatant was collected and filtered using a 0.22‐μm cellulose acetate syringe‐filter (GVS, Italy) and stored at ‐80°C until further use. EV isolation was performed using ExoQuick‐TC EV precipitation solution (System Biosciences, USA) according to the manufacturer's instructions. Cell culture media were centrifuged at 3000 × *g* for 15 min at 4°C. After centrifugation, the supernatant was filtered using a 0.22‐μm cellulose acetate syringe‐filter and mixed with ExoQuick‐TC solution. The mixture was kept at 4°C overnight and then centrifuged at 1500 × *g* for 30 min at 4°C. The EV pellet was dissolved in 1× PBS. EVs were stored at ‐80°C until further use.

### 2.2 SAP bead preparation and EV concentration

Two types of SAP beads were used. Poly(acrylamide‐co‐acrylic acid) SAP beads (CAS 9003‐06‐9, LG Chem Ltd., Korea) were purchased via TPY Co., Ltd., Korea (www.tpy21c.co.kr). Before use, the beads were washed three times continuously with ethanol and dried in an oven at 50°C. The average diameter and weight of single SAP bead investigated for EV concentration were 2.8 mm and 18 mg, respectively. SAP beads with average outer particle diameters were immersed in 1 mL of 1× PBS for up to 90 min to measure the absorbency of SAP beads. Poly(acrylamide‐co‐itaconic acid) SAP beads were synthesized using a milli‐fluidic system (Xie et al., 2016). The water phase was prepared by dissolving 180 g/L acrylamide (AM; Merck, Germany), 20 g/L itaconic acid (IA; Sigma‐Aldrich, USA), 2.2 g/L ammonium persulfate (Bio‐Rad, USA), and 4.0 g/L *N*,*N*'‐methylenebis(acrylamide) (Sigma‐Aldrich, USA) in deionized water. The synthesis of poly(acrylamide‐co‐itaconic acid) SAP beads includes chain initiation, propagation, and crosslinking. First, ammonium persulfate is decomposed at a high temperature (80°C) to produce sulfate anion radicals. Then, these anion radicals abstract the double bond of a monomer, which, in turn, reacts with another monomer to form a trimer. Finally, *N*,*N*'‐methylenebis(acrylamide) leads to the formation of cross‐linked poly(acrylamide‐co‐itaconic acid). To synthesize the beads, mineral oil (Sigma‐Aldrich, USA) was used as the oil phase. The water and oil phase flow in a T‐junction (Cowie, UK) with a 2 and 4.5 mm inner and outer diameter, respectively, were injected into a PFA tube with a 1/16‐inch inner diameter. The water‐in‐oil droplets were generated. Two high‐precision syringe pumps (NE1000_syringe Pump; New Era Pump Systems Inc., USA) were used to inject the oil and water phases at 5.0 mL/min and 10.0 mL/min, respectively, to generate 25 μL water phase droplets. The monomers inside the droplets were polymerized in the tube for 4 h at 80°C. Synthesized beads were separated from the mineral oil and washed using deionized water and ethanol, followed by drying under a vacuum at 40°C overnight. The surface morphology of SAP beads was examined under scanning electron microscope (SEM). SAP beads were coated with a thin layer of pure platinum and imaged in SEM (Model JSM‐7001F; JEOL Ltd., Japan), with an operating voltage of 15.0 kV. The EV concentration in the PBS solution was evaluated by immersing 450 mg/mL SAP beads in 1 mL of EV solution with 1.0 × 10^10^ EV particles.

### 2.3 Quantification of EV particles and total protein contents

The number and sizes of EVs were measured by a nanoparticle tracking analysis (NTA, threshold 4, time 30 s, frame particles < 100) using the Nanosight NS300 system (Malvern, UK). Camera focus was adjusted to visualize the sharp individual dots. The total protein concentration was measured by a BCA assay (Thermo Scientific, USA) or Bradford assay (Sigma‐Aldrich, USA). The BCA working reagent was prepared following the manufacturer's protocol. Each unknown sample and standard were diluted and mixed with the reagent solution, respectively, and incubated at 37°C for 30 min. The absorbance was measured at 562 nm using a spectrophotometer. The same experimental settings were used for measuring the EV and protein concentrations among the batches. The Bradford working reagent was prepared following the manufacturer's protocol. Each unknown sample and standard were diluted and mixed with the reagent solution, respectively, and incubated at room temperature for 10 min. The absorbance was measured at 595 nm using a spectrophotometer. The same experimental settings were used for measuring the EV and protein concentrations among the batches.

### 2.4 Western blot analysis, enzyme‐linked immunosorbent assay (ELISA), transmission electron microscopy (TEM) and dynamic light scattering (DLS) analysis

SDS‐PAGE was performed with reducing (TSG101, Syntenin, Hsc70, Calnexin, GM130) or non‐reducing (CD63, Tamm‐Horsfall protein (THP)) conditions. The same number of EV particles (2 × 10^9^ particles) was used for the comparison. A western blot analysis was performed with primary antibodies including mouse anti‐TSG101 (Abcam, ab83, UK) at 1:1000, mouse anti‐CD63 (MBL, MEX002‐3, USA) at 1:1000, rabbit anti‐Syntenin (Abcam, ab133267, UK) at 1:2000, rabbit anti‐Hsc70 (Abcam, ab51052, UK) at 1:500, rabbit anti‐GM130 (Abcam, ab52649, UK) at 1:1000, rabbit anti‐Calnexin (Cell Signaling Technology, 2679S, USA) at 1:1000, and rabbit anti‐THP (UMOD; Abcam, ab207170, UK) at 1:1000. For the detection, horseradish peroxidase‐conjugated anti‐mouse (Abcam, ab6728, UK) at 1:2000 and anti‐rabbit IgG (Cell Signaling Technology, 7074S, USA) at 1:1000, and an enhanced chemiluminescence detection system (Bio‐Rad, USA) were used. Images were captured using a ChemiDoc XRS+ imaging system (Bio‐Rad, USA). For ELISA, EV concentrations were measured by ExoELISA Complete Kit (Cat. No. EXOEL‐CD63A‐1, System Biosciences, USA) using CD63 antibody following the manufacturer's instructions. For TEM, the sample was applied to the copper grids coated only with a thin carbon foil (Cat. No. 01340, Ted Pella, Inc. USA). After allowing the sample to absorb for 2 min and blotting off buffer solution onto Whatman paper, the sample on the grids were stained with 2% (w/v) uranyl acetate (UrAc) for 1 min. Then, distilled water was added for 1 min to blot off UrAc followed by drying for 15 min. The result was recorded with Bio‐High voltage EM system (JEM‐1400 Plus at 120 kV and JEM‐1000BEF at 1000 kV; JEOL Ltd., Japan) at the Korea Basic Science Institute in the Republic of Korea. The zeta potential and polydispersity index (PDI) of the EVs were measured by DLS (Zetasizer NS, Malvern, UK) at 25°C. The same EV concentrations were adjusted to the equal volume in disposable folded capillary cell (Cat. No. DTS1070, Malvern, UK) and solvent resistant micro cuvette (Cat. No. ZEN0040, Malvern, UK) for measuring zeta potential and PDI, respectively.

### 2.5 Collection and pretreatment of human urine samples

First urine of healthy fasting donors between the ages of 20 and 50 years was collected in the morning before eating, smoking and drinking. The studies were approved by the Incheon National University Institutional Review Board (IRB 7007971‐2020001‐001‐01A). Urine samples were collected in a sterilized specimen cup (SPL Life Sciences, Korea) and stored at ‐80°C until further use. The characterizations of liquid samples including salt concentration and pH are listed in Table S1. For pretreatment, urine samples were subjected to centrifugation at 300 × *g* for 10 min to remove cells, and 3000 × *g* for 20 min and 17,000 × *g* for 20 min to remove cell debris, bacteria and other proteins. All centrifugation steps were performed at 4°C. Pretreated urine samples were filtered using 0.22‐μm cellulose acetate syringe‐filter.

### 2.6 Sample concentration by ultrafiltration or SAP beads and EV isolation by size exclusion chromatography (SEC)

To compare the concentration performance of ultrafiltration and SAP beads, cell culture media or pretreated human urine samples were concentrated first by ultrafiltration or SAP beads, followed by concentrated EV isolation by SEC. HeLa cells were grown in an EV‐free medium containing FBS. The medium (12 mL) was centrifuged at 3000 × *g* for 15 min to remove the debris and filtered using 0.22‐μm cellulose acetate syringe‐filter. The Amicon Ultra‐15 Centrifugal Filter Unit with a 10 kDa pore (Millipore, USA) was used for ultrafiltration, and cell culture media grown in EV‐free FBS were centrifuged at 4000 × *g* for 120 min at 4°C (90 min for pretreated urine). Additionally, SAP beads were used for concentrating cell culture media or urine samples at 250 mg/mL for 30 min. The supernatant containing EVs was collected. For SEC, 0.5 mL of the concentrated sample was coated on a qEV size exclusion column (Izon Science, New Zealand) and eluted with PBS; 0.5 mL of each fraction was collected in a microcentrifuge tube (SPL Life Sciences, Korea), and isolated EVs were used immediately or stored at ‐80°C until further use.

### 2.7 SAP bead‐based EV concentration and miRNA detection using a molecular beacon (MB)

An MB was designed to target mature miR‐21 (MB‐21). The MB‐21 sequence used in this study was 5′‐Cy3‐GCGCGTCAACATCAGTCTGATAAGCTACGCGC‐BHQ2‐3′ (Cho et al., 2019; Lee et al., 2015, 2016, 2018). MB‐21 with cyanine‐3 (Cy3) was designed using the UNAFold tool provided by Integrated DNA Technologies (IDT, USA) and synthesized IDT. To evaluate the specificity, MB‐21 and miR‐21 were mixed and incubated at 37°C for 1 h. Hybridization of MB‐21 with miR‐21 or with miR‐21 in EVs was assessed using a Varioskan Flash Multimode Reader (Thermo Scientific, USA). The excitation and emission wavelengths were 545 nm and 570 nm, respectively. For EV miR‐21 detection in the EV samples concentrated using SAP beads, 200 mg/mL SAP beads were incubated with the EV‐containing solution and incubated for 30 min for concentration. The supernatant was collected, and the detection signals were measured using a Varioskan Flash Multimode Reader.

### 2.8 Statistical analyses

Paired *t*‐tests were performed using GraphPad Prism 7 (GraphPad Software, La Jolla, CA, USA) to assess differences between two groups. Shapiro‐Wilk test was performed to assess normality distribution before using paired t‐tests.

## 3 RESULTS AND DISCUSSION

### 3.1 Time‐ and concentration‐dependent volume reduction by SAP

Water absorption by SAP beads in a solution causes bead swelling. As shown in Figure 1b, SAP bead swelling was observed after incubation. The bead size increased dramatically after incubation, while the solution volume decreased. To evaluate the effectiveness of SAP beads for EV concentration, time‐ and concentration‐dependent volume reduction properties were assessed. First, a single SAP bead was added to 1 mL of PBS solution and incubated for up to 90 min. The solution volume decreased drastically as the incubation time increased, and saturation began 80 min after incubation (Figure 1c). The volume decreased to 89.8% by a single bead at 30 min after incubation. Based on these results, SAP concentration‐dependent volume reduction was analyzed at 30 min after incubation (Figure 1d). Different concentrations of SAP were tested for absorption profiles. SAP beads gradually absorbed the solution, and the solution volume decreased to 10.0% using 200 mg/mL SAP beads. The average water absorption rate per SAP bead within the first 30 min was 3.32 μL/min and that after 30 to 90 min was 1.68 μL/min. To investigate the effect of ionic strength on water absorption by SAP beads, the SAP beads were incubated with the solution containing different NaCl concentrations for 30 min. As shown in Figure S1A, even the volume of 500 mM NaCl solution decreased to 10.4% when using 300 mg/mL SAP beads. In addition, pH did not alter the absorption efficiency of the SAP beads (Figure S1B). These results indicate that SAP effectively absorbed water molecules, and the degree of absorption can be easily controlled by adjusting the absorption time and SAP concentration. The solution can be successfully concentrated using a higher concentration of beads or longer incubation time. Herein, 200 mg/mL SAP beads that enabled 10‐fold volume reduction within 30 min were selected to investigate EV concentration in PBS solution.

### 3.2 Single‐step equipment‐free EV concentration using SAP beads

**FIGURE 2:**
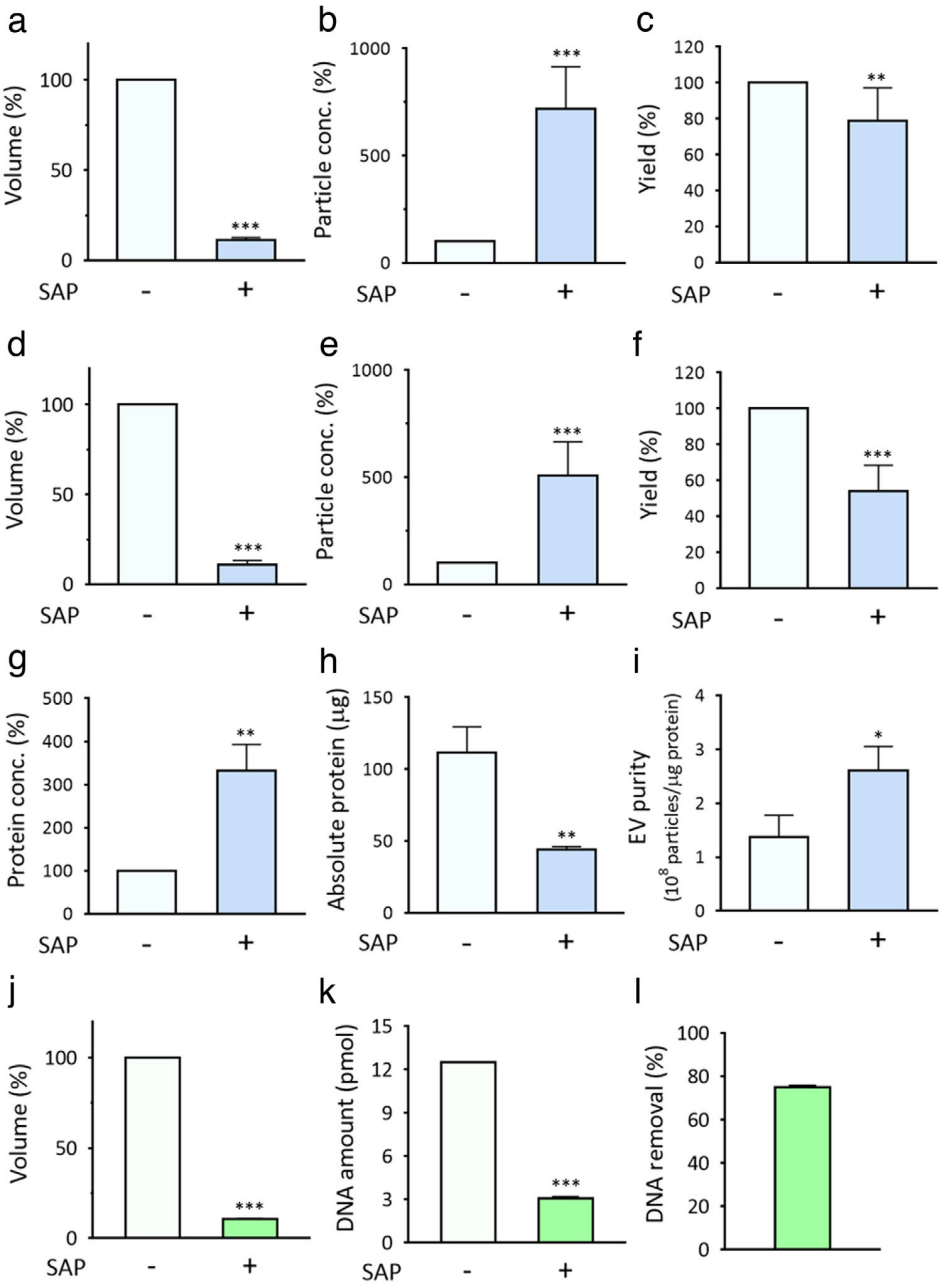
EV concentration, and protein and DNA impurity removal by SAP beads. [(a) HeLa cell‐derived EVs purified by ExoQuick‐TC were concentrated by the addition of 200 mg/mL SAP beads to an EV‐containing solution. The reduced volume was measured, and the % volume reduction was calculated 30 min after incubation. (b) Increase in the EV concentration after 30 min of EV enrichment. (c) The EV yield was calculated based on the remaining volume and particle concentration. (d‐e) Purer EVs purified by SEC were tested for EV concentration. (d) EVs were concentrated by the addition of 200 mg/mL SAP beads to an EV‐containing solution. The reduced volume was measured, and the % volume reduction was calculated 30 min after incubation. (e) Increase in the particle concentration after 30 min of EV enrichment. (f) The EV yield was calculated based on the remaining volume and particle concentration. (g) EVs purified by ExoQuick‐TC were concentrated by the addition of 200 mg/mL SAP beads to an EV‐containing solution. Protein concentrations before and after SAP bead addition were assessed. (h) Absolute protein amounts were measured based on the remaining volume and protein concentration. (i) EV purities (10^8^ particles/μg protein) in the EV solution before and after incubation with SAP beads. (j‐l) To demonstrate nucleic acid impurities can be also removed by SAP beads, oligonucleotide DNA (5′‐CCG CGT CAA CAT CAG TCT GAT AAG CTA CGC GG‐3′) with FAM dye was spiked into PBS solution and incubated with 200 mg/mL SAP beads. (j) The volume was measured 30 min after incubation. (k) Absolute DNA amount before and after SAP bead addition were assessed. (l) DNA removal efficiency was calculated. All values are presented as means ± SD (*P < 0.05, **P < 0.01, ***P < 0.001; n = 3∼10)]

To demonstrate that SAP beads absorb small molecules, including water, but not EVs, EVs were isolated from the culture medium by ExoQuick‐TC and spiked into PBS. The medium was filtered with 0.2‐μm filters before EV isolation because the primary purpose of this method is to concentrate EVs with a size less than 200 nm that are hard to concentrate. Then, the EV‐containing solution was incubated with 200 mg/mL SAP beads (11 SAP beads) for 30 min and the EV concentration and volume were measured. As shown in Figure 2a, the volume of the EV‐containing solution decreased to 11.1% after absorption. The EV‐concentrated solution was obtained. As determined by NTA, as compared to the initial EV concentration (1.2 × 10^10^ particles/mL), the final EV concentration after SAP incubation was 8.27 × 10^10^ particles/mL. With a single‐step and simple incubation process, the EV concentration reached approximately 720% as compared to that of original solution (Figure 2b). This can be explained by the small size of the internal SAP bead channel, which is less than several nanometers (Xie et al., 2016). Exosomes, which are the smallest EVs, are generally larger than 50 nm; accordingly, most EVs can be retained in the residual non‐absorbed solution. The final yield of EVs after concentration was 78.9%, calculated by dividing the concentrated EV count by the initial EV count (Figure 2c). The yield can be further improved by designing and constructing a simple device that can recover the residual solution among the beads after concentration. To rule out the effect of remaining polyethylene glycol from ExoQuick‐TC used for the preparation of EVs, we also used purer EVs isolated by SEC (Figure 2d‐f) and investigated concentration by SAP beads. Significantly lower levels of protein impurities (24‐fold) were included in the solution containing EVs isolated by SEC (Figure S2A). NTA (Figure S2B) and western blot analysis performed to determine the levels of the markers in cells and EV (Figure S2C) demonstrated the isolation of EVs. As shown in Figure 2e, the EV concentration reached approximately 510% as compared to that of the original solution with a single‐step incubation process (*P < 0.05, **P < 0.01, ***P < 0.001; n = 3∼10). Thus, the method can be applied for the concentration of EVs regardless of the isolation method.

The newly developed concentration method is beneficial for various biomedical applications of EVs. For instance, the large‐scale production of EVs is required for EV‐based therapeutic drug development owing to the high dose needed for treatment. A high EV concentration also improves the probability of biomarker detection from EVs. The conventional method including ultrafiltration (UF) can be used for concentrating EVs. However, they require a centrifuge and filters that increase the time, labour and the cost of production. Thus, these methods are inappropriate for large‐scale production or high throughput analyses of EVs. In contrast, our SAP bead‐based concentration method does not require any equipment for concentration and is extremely low cost. Thus, the SAP bead‐based EV concentration method offers great advantages over conventional concentration methods, including UF.

### 3.3 Removal of protein impurities during concentration by SAP beads

Many EV isolation methods are limited by impurities, including proteins, originating from the samples. This is a major issue because it results in a low purity of the isolated EV solution. For instance, EV isolation from cell culture medium results in a high concentration of protein impurities, including bovine serum albumin, from foetal bovine serum (Lobb et al., 2015; Webber & Clayton, 2013). This also applies to EV isolation from human plasma or serum samples, which are enriched with circulating protein impurities other than EVs. These impurities should be further reduced to ensure a high diagnostic accuracy in liquid biopsy and to minimize side effects, including toxicity and immunogenicity, when EVs are injected into humans as therapeutic drugs (Gudbergsson et al., 2019).

Although the main purpose of our method is EV enrichment and not EV purification, we hypothesized that SAP beads might also absorb protein impurities from the solution because many proteins are within several nanometers in size. To evaluate this hypothesis, protein concentrations before and after EV enrichment were measured. The same experimental conditions used for EV concentration were used for the analysis. First, the protein content increased 333% after SAP bead concentration, as expected based on the substantial reduction in solution volume by SAP beads (Figure 2g). However, surprisingly, considering the volume change of the solution before and after concentration, the absolute amounts of protein impurities were 112 μg and 44.3 μg, respectively (Figure 2h). To rule out the potential effect of lipid‐dye interaction in the protein quantification using BCA assay, the protein amount was also measured using the Bradford assay. As shown in Figure S3, the absolute amounts of protein impurities before and after concentration were 132.8 μg and 15.8 μg, respectively, consistent with the result in Figure 2h. These results clearly show that some protein impurities were absorbed by SAP beads and removed from the EV‐concentrated solution. Consequently, the purity of the EV solution (number of EV particles/μg protein) increased significantly from 1.38 to 2.61 × 10^8^ particles/μg protein, equivalent to a 190% increase in purity (Figure 2i). To directly prove the absorption of protein impurities by SAP beads during concentration, 6 × 10^9^ particles/mL EVs isolated by SEC were added to the 500 μL PBS containing different concentrations of BSA (0, 0.2, and 1 mg/mL) followed by SAP bead addition. After 30 min of concentration, volume, EV concentration and protein concentration were measured. As shown in Figure S4A and B, there were no significant differences of the volume reduction and EV enrichment regardless of BSA concentration. Interestingly, the absolute protein concentration mainly from BSA was drastically decreased meaning that BSA was absorbed to the SAP bead during concentration (Figure S4C). Thus, SAP beads absorbed some of the protein impurities during EV concentration and thereby increased the purity of solution. Thus, SAP beads absorbed the protein impurities and thereby increased the purity of solution. Of note, impurity removal during EV concentration can broaden the options for EV isolation, especially when the isolation method does not result in a high purity. The levels of small molecules other than proteins are also expected to be reduced by the same mode of action. To verify this, we incubated 25 nM FAM‐labelled oligonucleotide DNA (32 bp) with SAP beads for 30 min and assessed the volume reduction (Figure 2j), absolute DNA amount (Figure 2k), and DNA removal efficiency (Figure 2l). Significant amount of DNA was cleared away from the solution by SAP beads. Greater than 75% of DNA were removed and absorbed by SAP beads. Oligonucleotide DNA with different fluorescence dye (Cy3) and DNA sequence showed the similar results meaning the removal was not fluorescence dye and sequence‐dependent (Figure S5A‐C). Thus, ability of SAP beads to remove impurities is not limited to proteins but can be expanded to various molecules, including nucleic acids, chemicals, and lipids.

To explore the size‐dependent absorption of molecules by SAP beads, we tested if SAP beads can absorb doxorubicin, polystyrene microspheres and gold nanoparticles with the diameters of 1.5, 30 and 45 nm, respectively. Each material was incubated with the same amount of SAP beads for 30 min, and the SAP beads were separated and observed. As shown in Figure S6, the SAP beads incubated with doxorubicin were coloured red, indicating that high levels of doxorubicin were absorbed by the SAP beads. In contrast, SAP beads incubated with red‐coloured polystyrene microspheres and gold nanoparticles showed clear and transparent images, indicating that they were not absorbed by the beads. We can infer from these results that SAP beads can selectively absorb small materials, including water and impurities, thereby concentrating larger materials, including EVs. Because each material's properties other than size may also affect the absorption to SAP bead, further investigation of the physical and biochemical properties of materials that facilitate the absorption to SAP bead may contribute to the wide application of this method. Accordingly, the SAP bead‐based EV concentration method has multiple benefits, yielding EV solutions with high concentration and low impurities.

### 3.4 EV concentration using poly(acrylamide‐co‐itaconic acid) SAP beads

**FIGURE 3:**
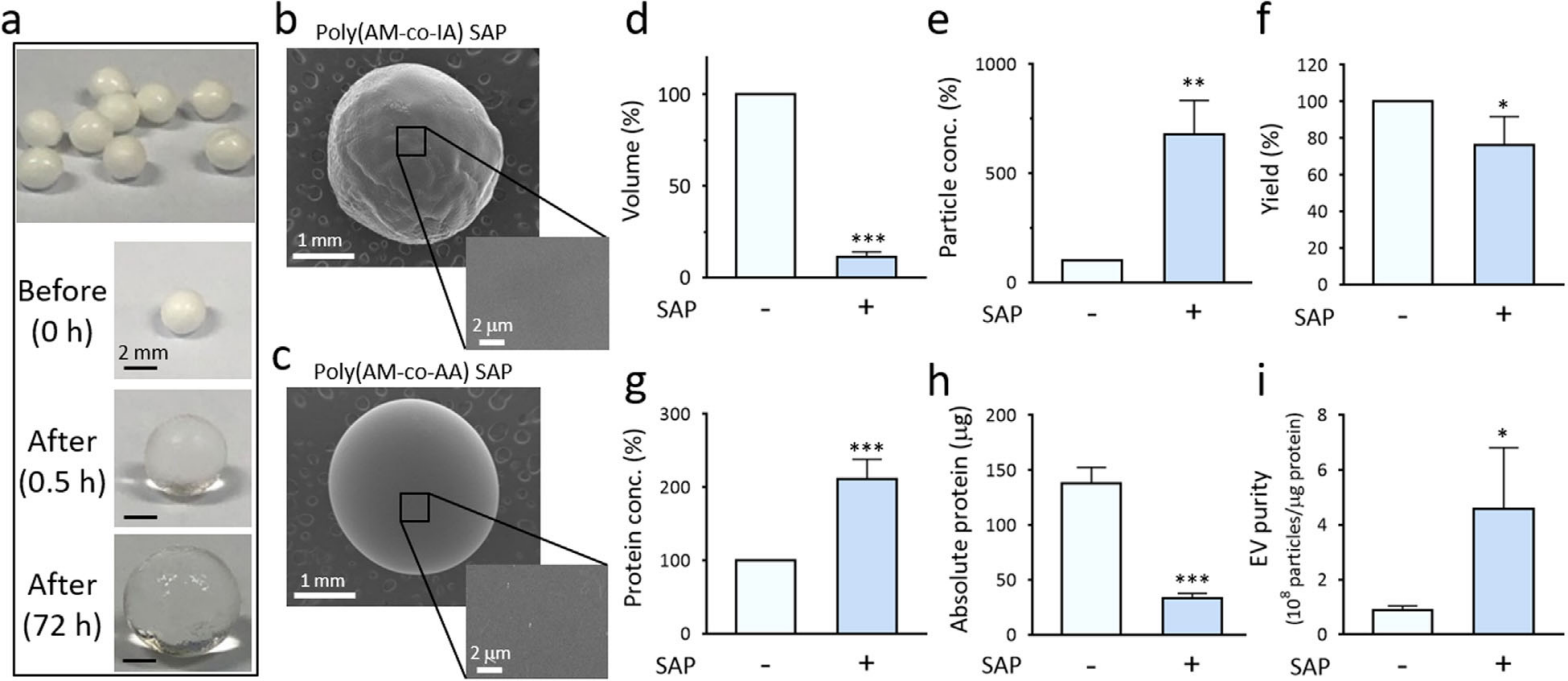
Synthesis of SAP beads and concentration of EVs. [(a) Poly(acrylamide‐co‐itaconic acid) SAP beads were synthesized. SAP beads were incubated in the solution for 0.5 to 72 h, and the images were obtained to show bead swelling. (b‐c) SEM micrographs of poly(acrylamide‐co‐itaconic acid) SAP bead (b) and poly(acrylamide‐co‐acrylic acid) SAP bead (c). Wide‐field and close‐up images showed the surface texture of each bead. (d) HeLa cell‐derived EVs purified by ExoQuick‐TC were concentrated by the addition of 450 mg/mL SAP beads for 30 min, and the volume was measured. (e) The increase in the particle concentration after enrichment was measured. (f) The EV yield was calculated. (g) Protein concentrations before and after SAP bead addition were assessed. (h) Absolute protein amounts were measured by using the remnant volume and protein concentration. (i) EV purities (10^8^ particles/μg protein) in the EV solution before and after SAP bead incubation. All values are presented as means ± SD (*P < 0.05, **P < 0.01, ***P < 0.001; n = 5)]

To determine whether other types of SAP beads with different chemical compositions have a similar EV concentration ability, poly(acrylamide‐co‐itaconic acid) SAP beads were synthesized (Figure 3a). The fabricated SAP bead presented a spherical form with some wrinkles on its surface which was probably caused by the drying process (Figure 3b). Similar to the poly(acrylamide‐co‐acrylic acid) bead (Figure 3c), the poly(acrylamide‐co‐itaconic acid) SAP bead exhibited a dense smooth surface with little to no visible porosity. Poly(acrylamide‐co‐itaconic acid) SAP bead was evaluated using the same EV‐containing solution used for poly(acrylamide‐co‐acrylic acid) beads. The volume of the EV‐containing solution decreased drastically to 11.4% and the EV concentration increased to 680% (Figure 3D, 3E). The yield after concentration was 76.0% (Figure 3f), indicating that poly(acrylamide‐co‐itaconic acid) SAP beads were also effective for EV enrichment. The western blot analysis and ELISA performed to determine the level of the EV marker demonstrated the concentration of EVs (Figure S7C). These results suggest that SAP beads with different compositions also successfully enrich EVs if the size of the water channel is sufficiently small to exclude EVs. Interestingly, poly(acrylamide‐co‐itaconic acid) SAP beads showed more efficient impurity removal (Figure 3g‐i) than that of poly(acrylamide‐co‐acrylic acid) SAP beads (Figure 2G–2I). This is probably due to the larger size of the water channel, enabling the additional absorption of protein impurities. Thus, the method can be implemented using SAPs with different compositions, including different combinations of monomers and crosslinkers according to the purpose of EV concentration.

### 3.5 Effect of SAP bead‐based concentration on EV properties

**FIGURE 4:**
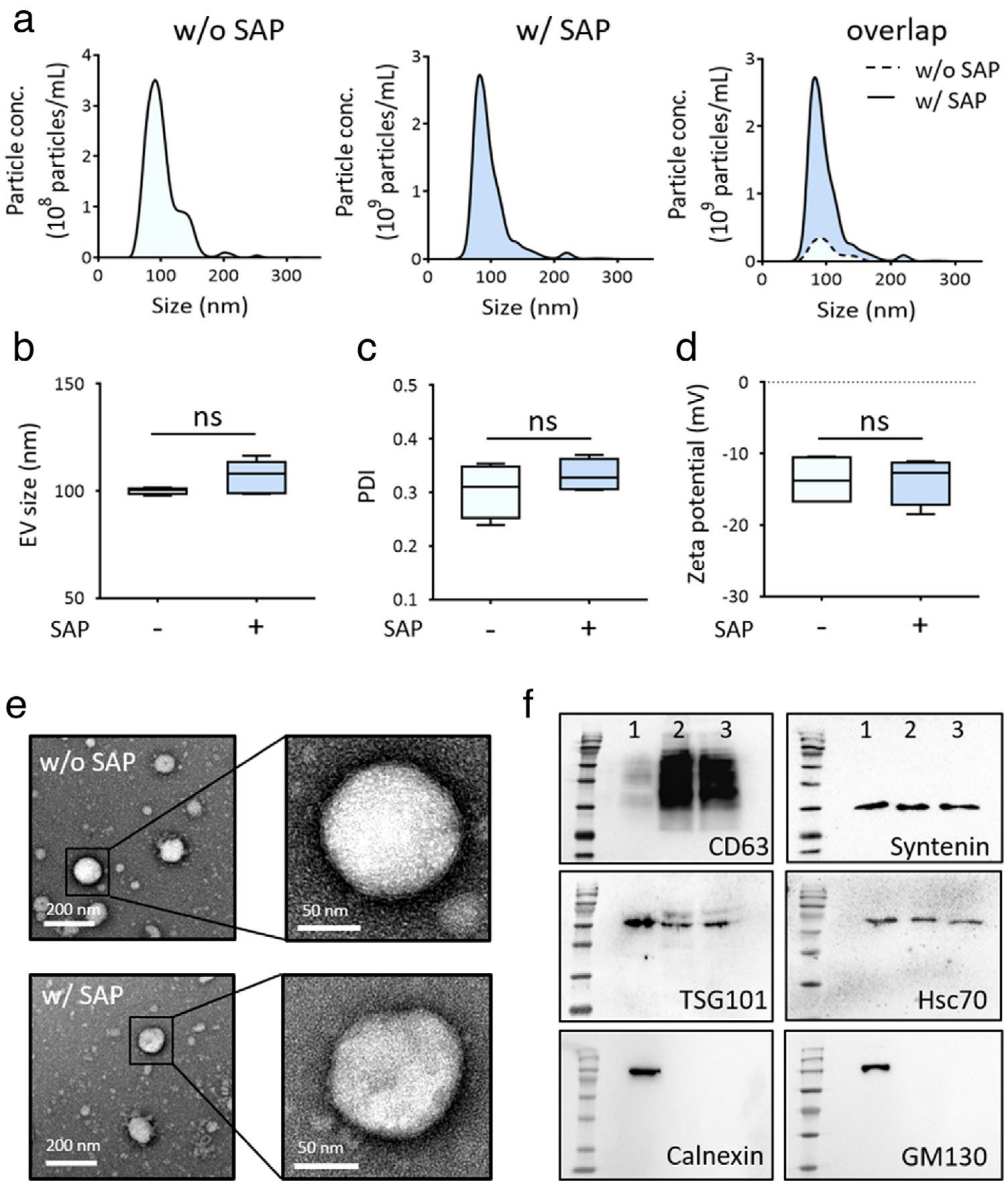
Characterization of EVs concentrated by SAP beads. [(a) Representative size distribution profiles of HeLa cell‐derived EVs analyzed by NTA before (w/o SAP) and after (w/ SAP) concentration by SAP beads. EVs purified by ExoQuick‐TC were used for the experiments. The size distribution profile of particles before concentration in the dotted box was enlarged. (b) The mean size distributions of particles before and after concentration by SAP beads were compared. No significant difference was observed. (c, d) Effect of SAP bead concentration on the PDI and zeta potential of EVs. (e) TEM images of samples before (left panel) and after (right panel) concentration. Close‐up images of EVs showed no apparent differences before and after concentration. (f) Western blot analysis of the marker proteins CD63, TSG101, Syntenin, Hsc70, Calnexin, and GM130. Lane 1: Cell, lane 2: unconcentrated EV, lane 3: concentrated EV. The same number of EV particles (2 × 10^9^ particles) was used for the comparison. The absence of Calnexin and GM130 in EV samples indicated no or little contamination of vesicles. All values are presented as means ± SD (ns: not significant; n = 5)]

For therapeutic or diagnostic applications of EVs concentrated by SAP beads, the concentration method should not affect the biochemical and biophysical properties of EVs. To evaluate whether the SAP bead‐based EV concentration method alters the physiological properties of EVs, the size, morphology, PDI, zeta potential and protein marker content of EVs were analyzed before and after enrichment. Based on NTA, there was no detectable difference in the size distribution of EVs before and after SAP bead concentration, despite a large difference in the EV concentration due to enrichment by SAP beads (Figure 4). The mean sizes of EVs were 100.1 nm before concentration and 106.6 nm after concentration, and the difference was not significant. Therefore, SAP bead concentration did not affect the size of EVs. Moreover, the SAP bead concentration method did not alter the dispersity and zeta potential of EVs (Figure 4c and d). TEM of EVs before and after concentration further showed that SAP beads did not alter the morphology of EVs (Figure 4e). A western blot analysis of CD63, TSG101, Syntenin, and Hsc70, representative EV protein markers, and Calnexin and GM130 were used to compare the protein contents of non‐concentrated and concentrated EVs (Figure 4f, S8). These analyses provide conclusive evidence that representative biochemical and physical properties of EVs are not altered by the enrichment process using SAP beads.

### 3.6 Concentration and isolation of EVs from human urine samples using SAP beads

**FIGURE 5:**
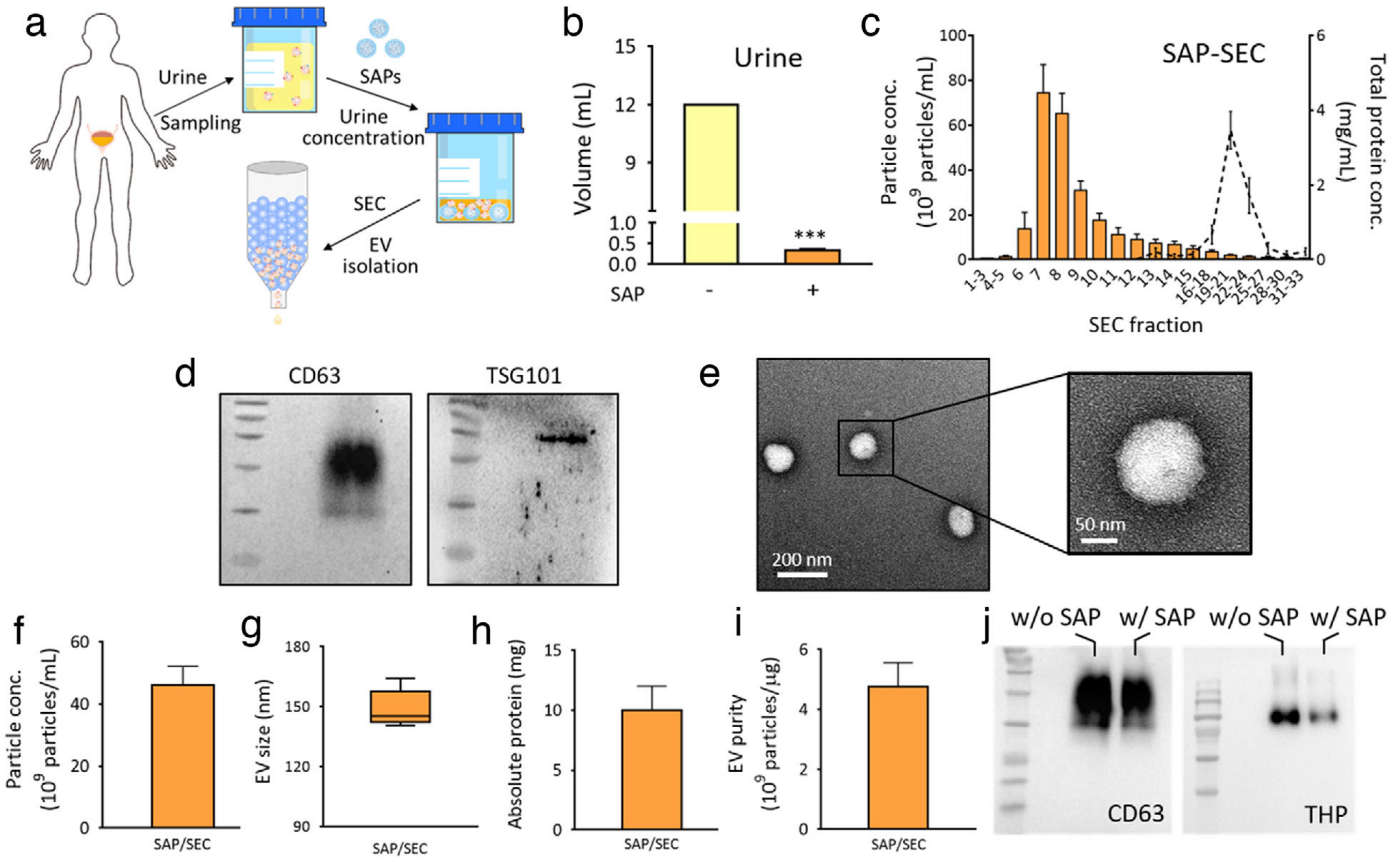
Size exclusion chromatography of EVs from concentrated human urine by SAP beads. [(a) Schematic illustration of EV isolation from human urine by SAP beads integrated with SEC is shown. (b) Concentration and volume reduction of human urine by SAP beads were assessed. (c) EV isolation using SEC from concentrated urine. Particle concentrations and total protein concentrations in all SEC fractions are shown. EV‐containing fractions (No. 6 to 9) were collected and used for further characterization otherwise mentioned. (d) Isolated EVs from urine was analyzed for the presence of EV markers, CD63 and TSG101 by western blot. (e) TEM image of isolated urinary EVs. (f–i) Average urinary EV concentration (f), size distributions (g), absolute protein in all fractions (h), and isolation purity (i) are shown. (j) Western blot analysis of THP in the human urine sample before (w/o SAP) and after (w/ SAP) concentration. After 10‐fold concentration, 7.5 μL and 0.75 μL of samples were used for comparison between urine samples w/o SAP and w/ SAP, respectively. Note that the absolute amount of CD63 (left) slightly decreased while THP (right) further decreased during concentration by SAP beads. All values are presented as means ± SD (***P < 0.001; n = 5)]

Owing to the easy and non‐invasive human urine collection, urinary EVs are attractive sources of biomarkers for liquid biopsy. Various strategies based on ultracentrifugation, precipitation, and SEC have been compared for EV isolation from healthy donors (Cho et al., 2020). SEC was the best method for EV isolation from human urine with respect to yield and purity (Benedikter et al., 2017; Nordin et al., 2015). However, the EV concentration in urine is lower than that in the blood, and it is necessary to concentrate urine before injection into the SEC column. Accordingly, we evaluated the use of SAP beads for the concentration of EVs in human urine, followed by SEC for EV isolation (Figure 5a). First, 12 mL of urine from healthy donors was concentrated with 250 mg/mL SAP beads. As shown in Figure 5b, the urine was drastically concentrated, and the volume reduced to 0.33 mL by SAP beads within 30 min (***P < 0.001; n = 5). The concentrated urine was further used for EV purification by injecting into an SEC column (Figure 5c). The main fractions (fractions 6–9) were collected and analyzed for EV yield, EV size and shape, EV marker, protein concentration and purity (Figure 5d‐i). EVs in concentrated urine were successfully isolated by SEC. Western blot analysis (Figure 5d) and TEM image of urinary EVs demonstrated the successful isolation of EVs from human urine (Figure 5e). Based on the results of EV purification by SEC column after urine concentration using UF (Figure S9), lower EV yield was obtained from urine concentrated by SAP beads. However, SAP bead‐based method has advantages over ultrafiltration considering the concentration time and cost. Also, the investigation of SAP bead composition and concentration condition optimal to human urine is required to increase the EV yield.

In addition, we also observed that SAP beads can absorb and remove some of the THP, a major contaminant urinary protein, during urine concentration. Western blot analysis of CD63 and THP levels in the urine before and after concentration by SAP beads showed that the absolute amount of THP (impurities) was drastically reduced. At the same time, there was a slight decrease in the absolute amount of CD63 (EVs), indicating that SAP beads removed a substantial amount of THP in the urine during EV concentration (Figure 5j). Overall, the results clearly demonstrated that the SAP bead‐based concentration method could be integrated with SEC for the efficient isolation of EVs from human urine. Considering the simplicity and low cost, SAP beads are a great alternative to other concentration methods.

### 3.7 Comparison of SAP and UF‐based concentration methods for EV purification from cell culture medium

For practical applications of EVs, large‐scale cell culture is necessary to achieve a high quantities of EVs from culture medium (Ramirez et al., 2018). A previous study has shown that EVs inhibit apoptosis in CHO cell culture, indicating that EVs have great potential as medium supplements to improve biological drug production (Han & Rhee, 2018). However, this requires the establishment of an efficient method for the enrichment of EVs produced by cells from large volumes of cell culture medium, thereby reducing the volume required for EV isolation.

**FIGURE 6:**
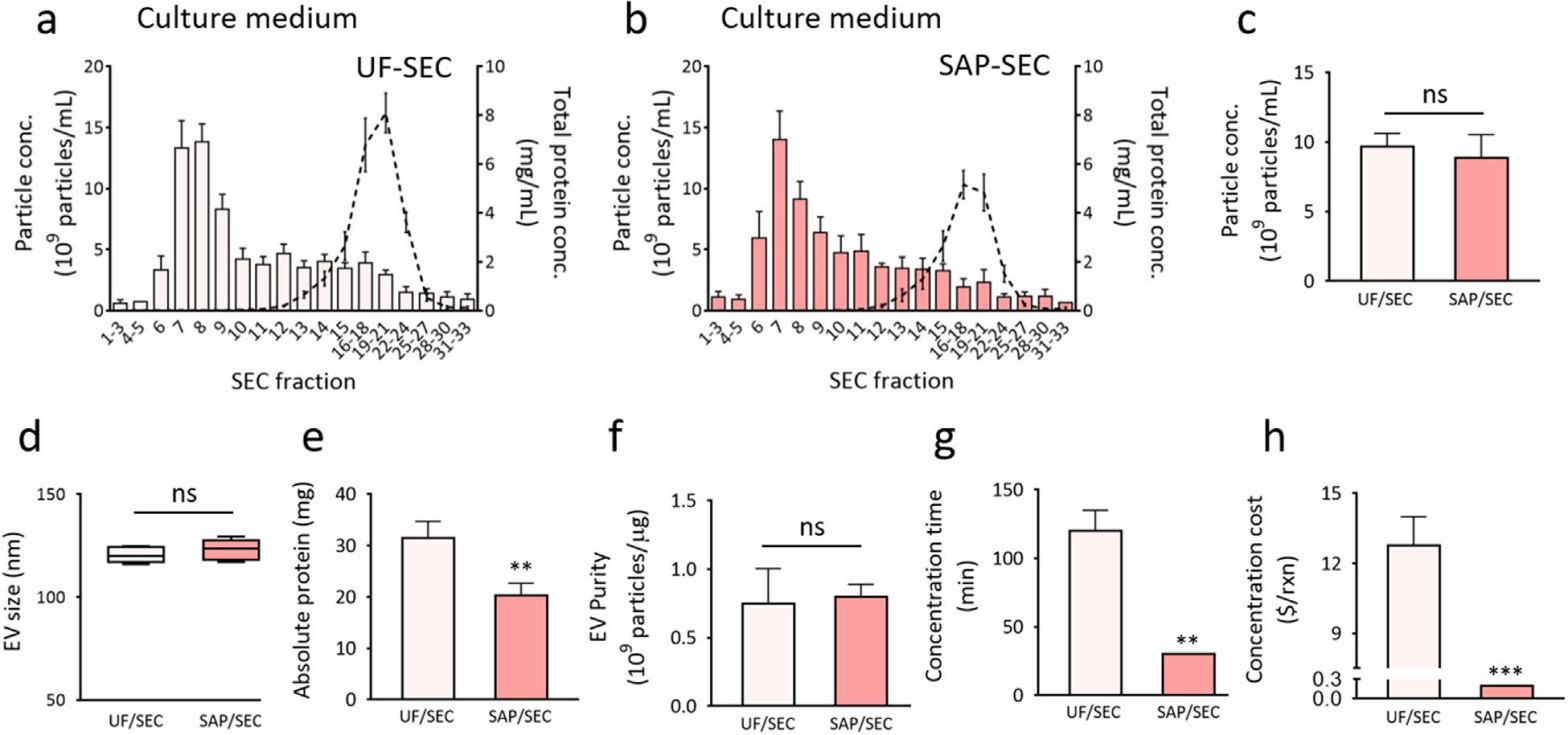
Isolation of EVs by SEC from culture media concentrated by UF or SAP beads. [(a, b) HeLa cell culture media were concentrated using UF (a) or SAP beads (b) followed by EV isolation by SEC. (c‐f) HeLa cell‐derived EV concentration and protein impurity profiles for each SEC fraction are shown. Isolated EV concentrations (c), average sizes (d), absolute protein amount (e), and EV purity (f) after SEC were compared between UF‐ and SAP bead‐based concentration methods. (g, h) The costs (g) and time (h) for a single concentration reaction of 15 mL of cell culture media were compared. Three commercially available UF filter membranes were evaluated. All values are presented as means ± SD (**P < 0.01, ***P < 0.001, ns: not significant; n = 5)]

To verify the versatility of our EV concentration method, SAP beads were used for EV enrichment from the cell culture medium. Recent studies have shown that the UF‐mediated concentration of EVs prior to EV isolation by SEC enables the purification of EVs from dilute cell culture media (Benedikter et al., 2017). To investigate whether SAP bead‐based EV concentration can be also used as an alternative to the UF‐mediated method, the same volume of cell culture media was used for EV enrichment using a membrane of 10 kDa pore size and SAP. Then, after EV concentration by the two methods, further isolation was performed by SEC. The EV yields and protein impurities in each separated fraction after SEC were compared between UF‐based or SAP bead‐based concentration methods (Figure 6a and b). The concentrations and average sizes of isolated EVs in the main SEC fractions (fractions 6–9) were almost the same in the concentrated culture medium obtained by each method (Figure 6c and d). Interestingly, the SAP bead‐based method showed a better protein impurity removal capability than that of the UF‐based method; the protein content was 1.55‐fold lower in the SAP bead‐based concentrated culture medium (**P < 0.01, ns: not significant; n = 5) (Figure 6e and f). The results indicate that the SAP bead‐based method can be successfully used for the concentration of EVs from the culture medium. We also investigated if the method is applicable for larger sample volumes and higher concentration folds. To demonstrate scalability, 250 mg/mL SAP beads were incubated with 100 mL of culture medium. As a result, the volume was reduced to less than 0.5 mL within 30 min, indicating that 200 times more potent concentrations can be achieved (Figure S10). Additionally, the time required for EV enrichment was generally much shorter and can be further reduced by increasing the SAP concentration (Figure 6g). Considering the average retail prices of consumable UF membranes (approximately $12.8 per UF reaction), as shown in Figure 6h, the SAP bead‐based method is highly cost‐effective, requiring only $0.2 per concentration reaction (1.57% the cost of UF). Overall, SAP bead‐based concentration of EVs offers many advantages and can be considered for large‐scale and effective isolation of EVs.

### 3.8 Application of the SAP bead‐based EV concentration method to EV biomarker detection

**FIGURE 7:**
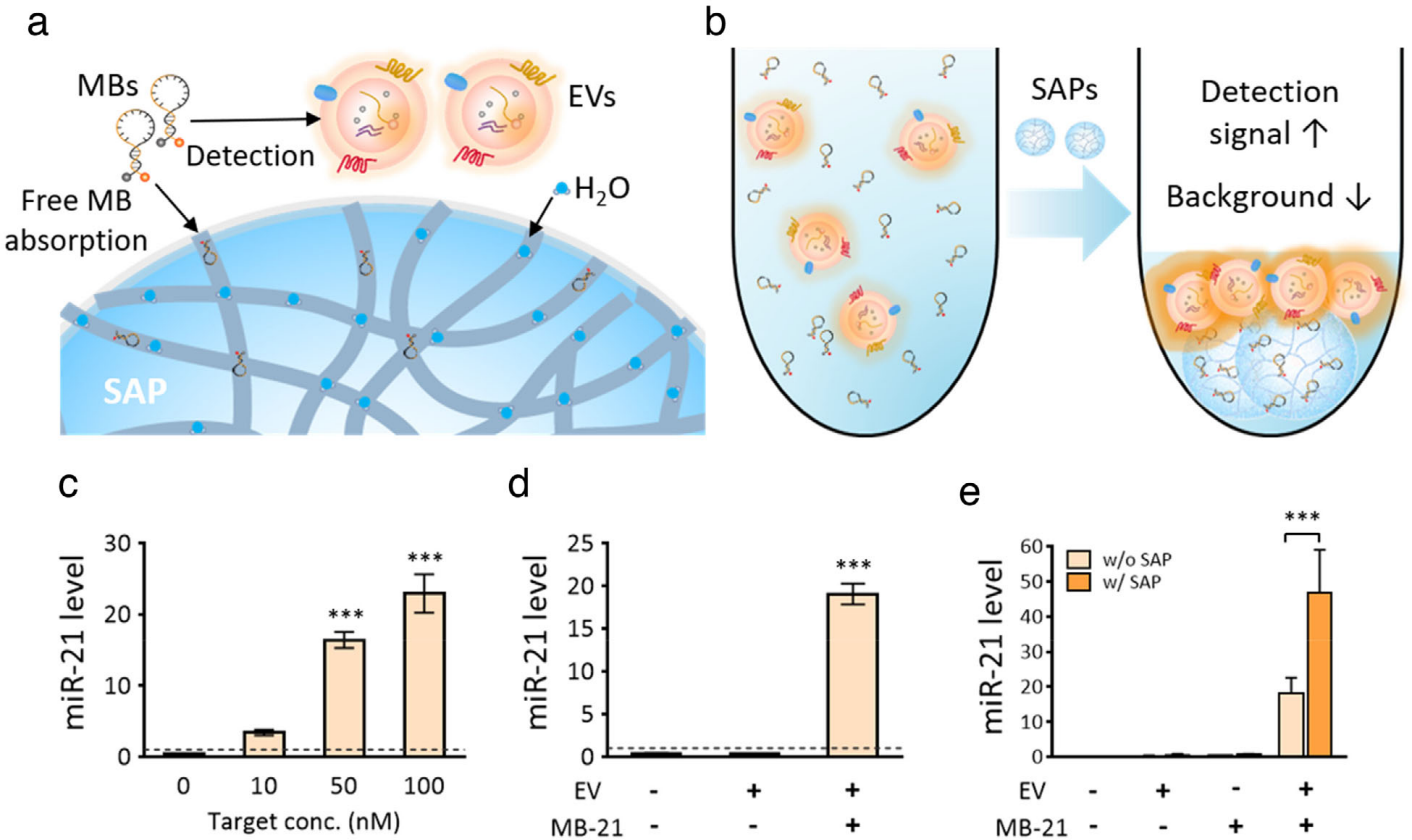
Application of EV concentration by SAP beads to EV miRNA biomarker detection. [(a, b) Free MB in the solution not involved in HeLa cell‐derived EV miRNA detection can be absorbed by SAP beads. This increases the detection signal by concentrating EVs and decreases the amount of free MBs in the solution. (c, d) Specific hybridization of MB‐21 to its target miR‐21 in the solution (c) and EVs (d). (e) SAP bead‐based EV miRNA detection using MB‐21. Detection signal of EV miR‐21 detection was significantly enhanced by SAP beads. EVs purified by ExoQuick‐TC were used for the experiments. All values are presented as means ± SD (***P < 0.001; n = 3)]

EVs, including exosomes, play important roles in the pathogenesis of diseases, such as cancer (Azmi et al., 2013), and the molecular content of EVs reflects specific physiological conditions of parental cells (Seigneuric et al., 2016). Thus, the development of an efficient method for detecting miRNAs in EVs is highly needed. In this context, we have recently developed a method for the *in situ* detection of miRNAs in exosomes in a single detection reaction using nano‐sized oligonucleotide probes, molecular beacons (MBs) (Cho et al., 2019; Lee et al., 2015, 2016, 2018). We have previously demonstrated the penetration of MBs and subsequent hybridization with target miRNAs into the EVs by treating the EVs with protease and RNase (Lee et al., 2015, 2018). The detection signal should increase, while the background signal from unbound free MBs should be minimized to improve the molecular detection of miRNAs in EVs. Owing to the size‐dependent absorption properties of SAP beads, unbound MBs can be absorbed by SAP beads due to their small sizes, while MBs penetrating EVs cannot (Figure 7a). At the same time, when EVs are highly concentrated by SAP beads, the detection signal from MBs and miRNA hybridization can be easily amplified. In this way, we can simultaneously increase the detection signal and decrease the background signal (Figure 7b).

To evaluate our hypothesis, an MB targeting miR‐21 (MB‐21) was constructed and used for miRNA detection in EVs. The specificity of MB‐21 was evaluated against synthetic miR‐21 and miR‐21 in EVs (Figure 7c and d). As shown in Figure 7c, the detection signal for MB‐21 and synthetic miR‐21 increased as the concentration of miR‐21 increased. Then, EVs were isolated from HeLa cells and incubated at various concentrations with MB‐21. The *in situ* detection signal increased significantly as the EV concentration increased. To improve the diagnostic ability of MB, we incubated an EV solution containing MB‐21 with SAP beads for EV concentration. The detection signal increased drastically from 18.2 to 46.7 by the addition of SAP beads (Figure 7e). In contrast, the background signal originating from free MB‐21 increased from 0.31 to 0.68. Thus, SAP beads absorbed the solution as well as some free MB‐21 during EV concentration, thereby providing good detection ability. This may substantially improve the detection sensitivity, especially when biomarker levels are low. The method is straightforward and simple, without requiring a complex process, materials, or device to increase the detection signal. The method is promising for the on‐site diagnosis as SAP bead enables the development of highly sensitive and portable diagnostic devices without any power supplies. The EVs cannot be separated from unbound probes using regular centrifuges, and the ultracentrifugation and polymer‐based precipitation of EVs result in significant loss of EVs. Thus, the reduction of unbound probes from the EV solution during concentration by SAP beads also contributes to minimizing the washing steps during the detection. The inner size of the SAP bead channel can be further optimized to maximize the absorption of the detection probe and minimize EV loss, making the SAP bead‐based EV concentration method widely applicable for the use of liquid biopsy samples in biomarker detection performed using various types of detection probes, including antibodies and aptamers. Adopting the same concept, this method is promising for drug delivery via drug‐loaded EVs, by the SAP‐bead mediated removal of therapeutic drugs undelivered to EVs.

### 3.9 Current challenges of SAP bead‐based EV concentration methods

Despite their broad and intensive uses in many fields, including agriculture and food industry, few studies have been conducted examining the application of SAP beads for biological purposes. Considering the potential use of SAP beads for the concentration of EVs shown in the study, further investigations should be conducted to overcome the current limitations. First, the SAP beads need to be carefully characterized when used primarily for the therapeutic EV preparations. Moreover, since the SAP beads used in this study were not originally constructed for the concentration of EVs in the solution, it was necessary to optimize their composition and synthetic conditions to adjust their absorption properties. Different mechanisms for the absorption of impurities by SAP beads may likely be involved. For instance, electrostatic interactions can occur between the SAP bead surface and proteins owing to the negative charges of the SAP beads. However, since the beads also absorbed the small oligonucleotide DNA, the electrostatic force was not the only way to introduce and absorb the impurities by beads. Based on this, it is speculated that the mechanism of impurity absorption depends on the type of impurities. Hence, depending on the type and size of impurities to be reduced during the concentration of EVs, SAP beads can be modified to accommodate a high level of impurities. For more practical and comfortable use of SAP beads for the concentration of EVs, the concentrated solution should be fully and easily recovered from the tube. In this context, the construction of an automated device that ensures easy separation of the concentrated solution from SAP beads will ensure convenient and user‐friendly operation. The EV‐containing solutions, including culture media and body fluids, are heterogeneous. No single method measures the full spectrum of EV characteristics, including an EV concentration, in those complex solutions. Currently used NTA measures all types of particles, including EVs, in the solutions. Thus, developing an advanced method or device that enables the specific and accurate quantification of EVs is required to quantify EV numbers in the concentration process using the SAP beads.

## 4 CONCLUSION

EVs are novel biomaterials widely applied in disease diagnosis and therapy. In accordance with their broad applications, remarkable EV isolation methods have been developed. However, methods for efficient EV concentration are lacking. Conventional ultrafiltration is costly and requires a long operation time and specialized equipment for EV concentration, emphasizing the importance of the development of a novel EV concentration method for large‐scale production and high‐throughput analyses. We demonstrated that SAP beads successfully enriched EVs in various solution types, including culture medium and human urine samples, and removed protein and nucleic acid impurities. The approach is simple, efficient, cost‐effective, scalable, and equipment‐free and can be widely used for therapeutic or diagnostic purposes requiring EV enrichment. This method may thus improve our ability to harness EVs for a variety of applications in diagnosis and therapy.

## CONFLICTS OF INTEREST

The authors declare that they have no conflict of interest.

## Supporting information

Supporting Information
