# Supplementary material for: Single‐step equipment‐free extracellular vesicle concentration using super absorbent polymer beads
Source: J Extracell Vesicles. 2021 Feb 23;10(4):e12074. doi: 10.1002/jev2.12074 (PMC7902527; doi:10.1002/jev2.12074)
Supplement: Supplementary file 1 — Supporting Information [file JEV2-10-e12074-s001.docx]

*Supplemental Materials*

**Single-step equipment-free extracellular vesicle concentration using super absorbent polymer beads**

**Hee Cheol Yang^1†^, Yoo Min Ham^1†^, Jeong Ah Kim^2, 3*^, and Won Jong Rhee^1, 4*^**

^1^ Department of Bioengineering and Nano-Bioengineering, Incheon National University, Incheon 22012, Republic of Korea.

^2^ Center for Scientific Instrumentation, Korea Basic Science Institute, Chungbuk 28119, Republic of Korea.

^3^ Department of Bio-Analytical Science, University of Science and Technology, Daejeon 34113, Republic of Korea

^4^ Division of Bioengineering, Incheon National University, Incheon 22012, Republic of Korea.

**^†^** Equal contribution

^*^ To whom correspondence should be addressed. Tel: +82 32 835 8299; Fax: +82 32 835 0763; Email: wjrhee@inu.ac.kr, jakim98@kbsi.re.kr

***Table S1.***

Table S1. Characterizations of liquid samples used for concentration experiments

| Samples | pH | Sodium (mM) | Potassium (mM) |
| --- | --- | --- | --- |
| Human urine | 5.9 | 103.1 ~ 157.4 ^[1,2]^ | 35~62.0 ^[1,2]^ |
| PBS solution | 7.4 | 157 | 4.50 |
| DMEM | 7.4 | 155.3 | 5.30 |

[1] Shahid.A, Quresbi. H, Nizami. F et al, Electrolytes in Liver Disease-A Preliminary Study, JPMA, 1983; 33: 289.

[2] Ali A. Ali, The Effect of Long Term use of Glibenclamide on Serum and Urinary Sodium and Potassium Level in Type 2 DM Patients, Iraqi J Pharm Sci, 2010; 19: 58-61.

***Figure S1***


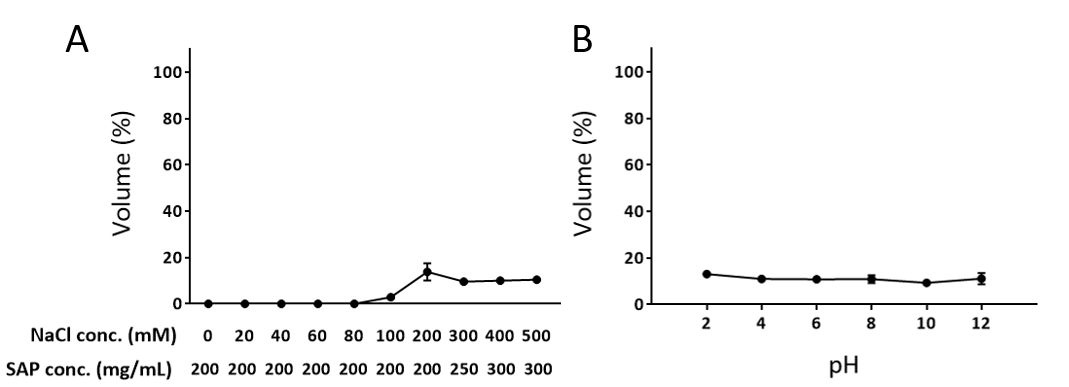


**Fig. S1. Effect of salt and pH on water absorption by SAP beads** (A) SAP beads were incubated with the solution containing different concentrations of NaCl for 30 min. The remaining volume percentage was calculated by measuring the remaining volume. (B) 200 mg/mL SAP beads were incubated in the solution with different pH for 30 min. The remaining volume percentage was calculated by measuring the remaining volume.

***Figure S2***


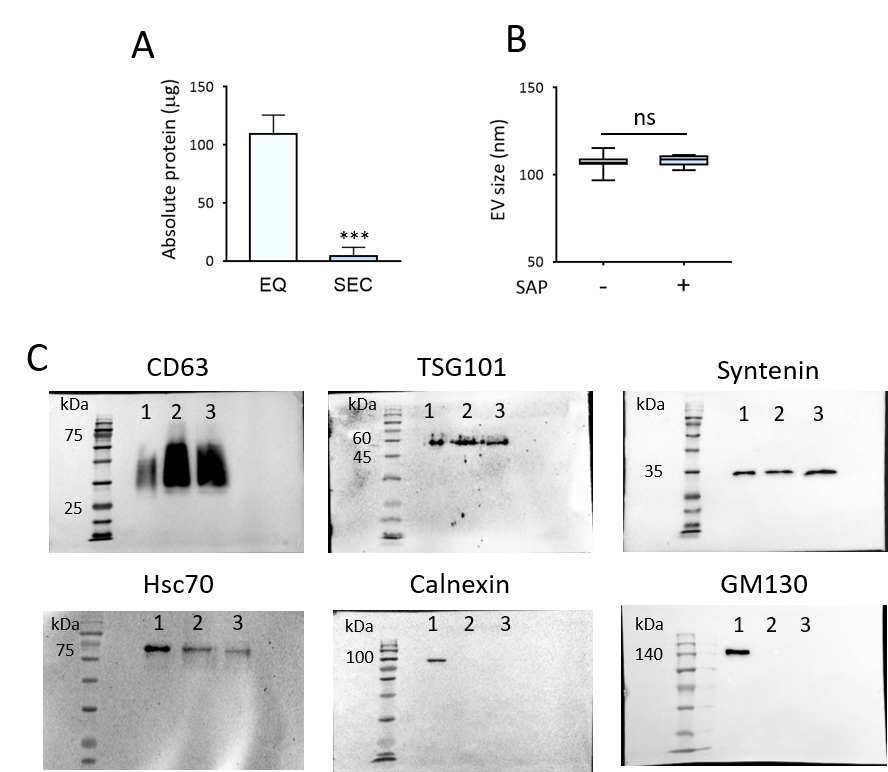


**Fig. S2. Characterization of concentrated EVs isolated by SEC** (A) HeLa cell-derived EVs were purified by SEC to generate purer EVs. The absolute protein amounts were compared between EVs isolated by ExoQuick-TC (EQ) and size exclusion chromatography (SEC). (B) The mean size distributions of EVs before and after concentration by 200 mg/mL SAP beads were compared. (C) Western blot analysis of the EV marker proteins in EVs isolated by SEC. Lane 1: Cell, lane 2: unconcentrated EV, lane 3: concentrated EV. The same number of EV particles (6.5 × 10^8^ particles) was loaded for comparison. All values are presented as means ± SD (***p < 0.001, ns: not significant; n = 3).

***Figure S3***

**
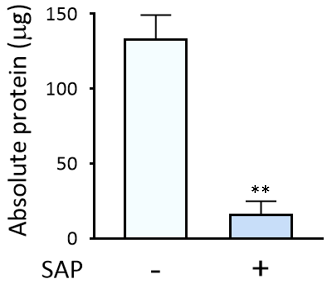
**

**Fig. S3. Protein amount measurement using Bradford assay.** HeLa cell-derived EVs isolated using ExoQuick-TC were concentrated by the addition of 200 mg/mL SAP beads to an EV-containing solution. Absolute protein amounts were measured based on the remaining volume and protein concentration using Bradford assay. All values are presented as means ± SD (**p <0.01; n = 4).

***Figure S4***

***
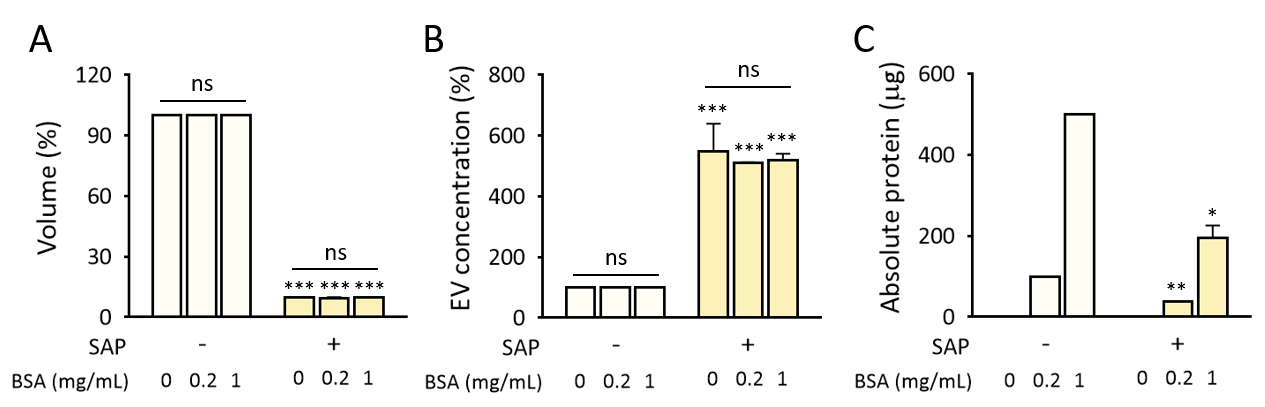
***

**Fig. S4. Concentration of EVs in BSA containing solution** Different concentrations of BSA (0, 0.2, 1 mg/mL) were added to PBS containing 6 × 10^9^ particles/mL EVs purified by SEC followed by SAP bead addition. (A) The reduced volume was measured, and the % volume reduction was calculated 30 min after incubation. (B) Increase in the EV concentration after 30 min of EV enrichment. (C) Absolute protein amounts were measured based on the remaining volume and protein concentration. The absolute protein amount decreased to 37.9 and 38.8% in the 0.2 and 1 mg/mL BSA-containing solutions, respectively. All values are presented as means ± SD (*p < 0.05, **p <0.01, ***p < 0.001, ns: not significant; n = 3).

***Figure S5***


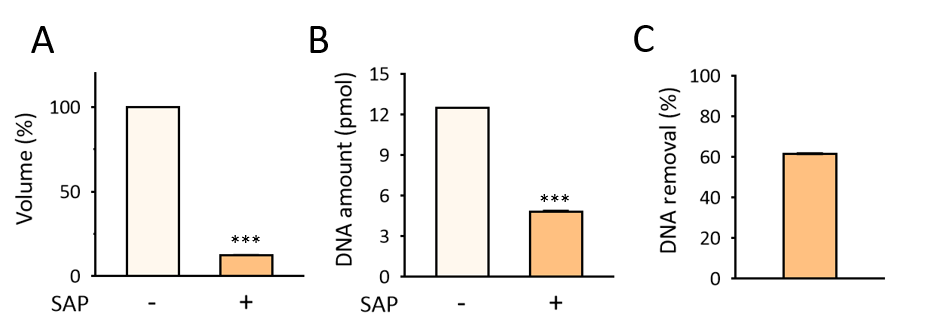


**Fig. S5. DNA impurity removal by SAP bead** (A) Cy3-labeled oligonucleotide DNA (5’-Cy3-GCG CGA CAA CAT CAG TCT GAT AAG CTA CGC GC-3’) was added to PBS solution and incubated with 200 mg/mL SAP beads. The volume was measured 30 min after incubation. (B) Absolute oligonucleotide DNA amount before and after SAP bead addition were assessed. (C) DNA removal efficiency was calculated. All values are presented as means ± SD (***p < 0.001; n = 3).

***Figure S6***


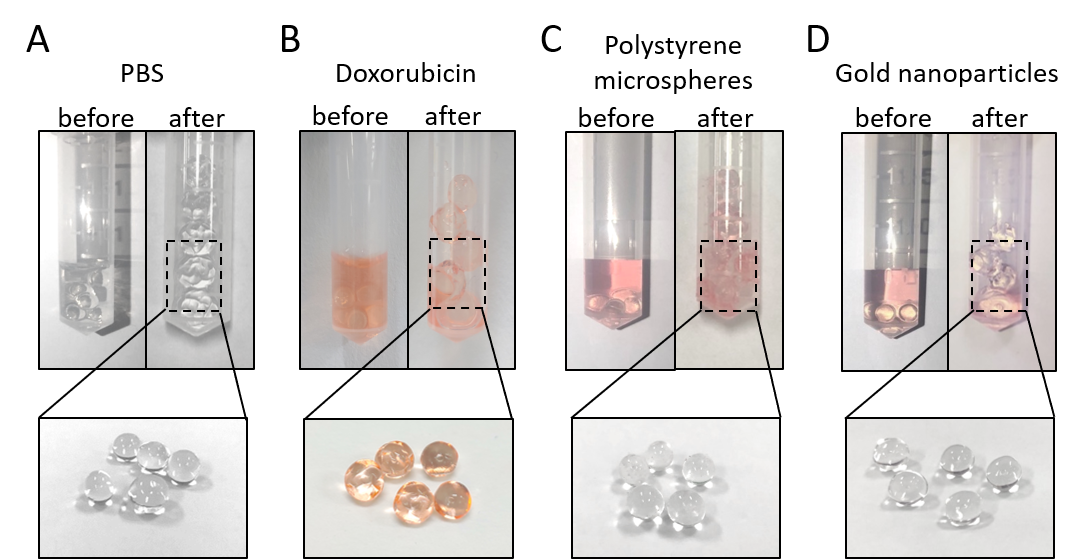


**Fig. S6. Size-dependent absorption by SAP beads** SAP beads were incubated with the solution containing (A) PBS only, (B) doxorubicin, (C) red colored polystyrene microspheres, and (D) gold nanoparticles, respectively, for 30 min. The beads were briefly rinsed and photographed.

***Figure S7***


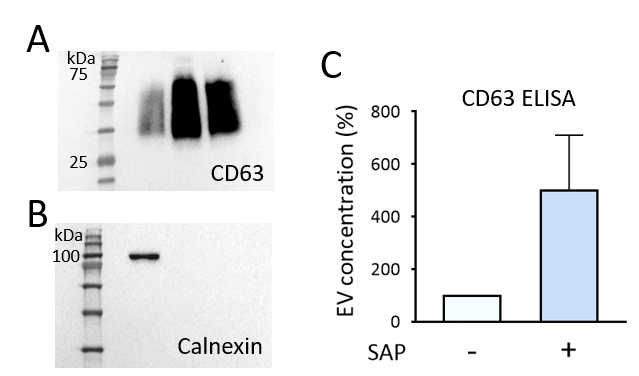


**Fig. S7. Western blot analysis and ELISA of samples concentrated using poly(acrylamide-co-itaconic acid) SAP bead** Western blot results of CD63 (A) and Calnexin (B). From the left, size marker, cell lysate, unconcentrated EVs, and concentrated EVs were loaded. The same number of EVs (2 × 10^9^ particles) was loaded for the comparison. For ELISA, the EV concentrations before and after EV enrichment were measured using CD63 antibody.

***Figure S8***


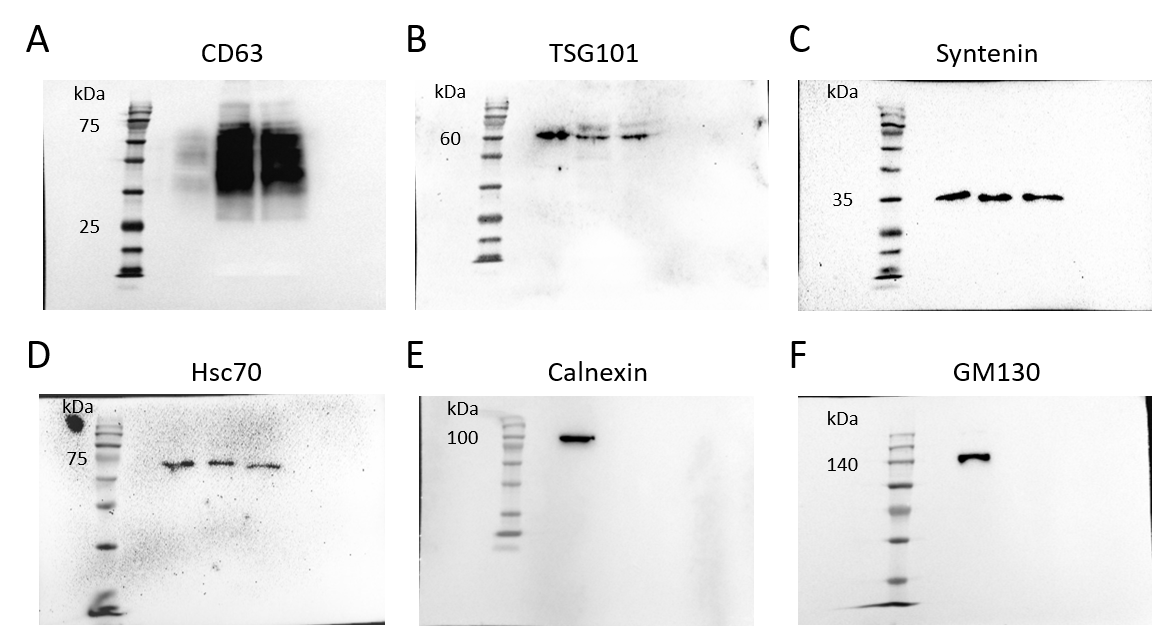


**Fig. S8. Western blot analysis of the EV marker proteins in the samples concentrated using poly(acrylamide-co-acrylic acid) SAP bead** Full blot results of CD63 (A), TSG101 (B), Syntenin (C), Hsc70 (D), Calnexin (E), and GM130 (F) shown in Fig. 4F. From the left, size marker, cell lysate, unconcentrated EVs, and concentrated EVs were loaded. The same number of HeLa cell-derived EVs (2 × 10^9^ particles) was loaded for the comparison.

***Figure S9***


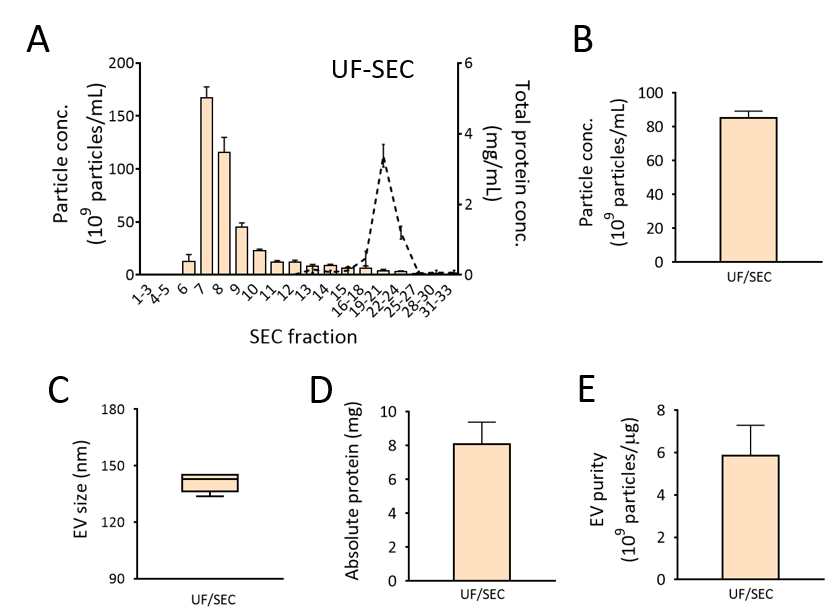


**Fig. S9. Size exclusion chromatography of EVs from concentrated human urine by UF** (A) Human urine was first concentrated using UF followed by EV isolation using SEC. EV concentrations and total protein concentrations in all SEC fractions were measured. Average urinary EV concentration (B), size distributions (C), absolute protein in all fractions (D), and isolation purity (E) are shown.

***Figure S10***


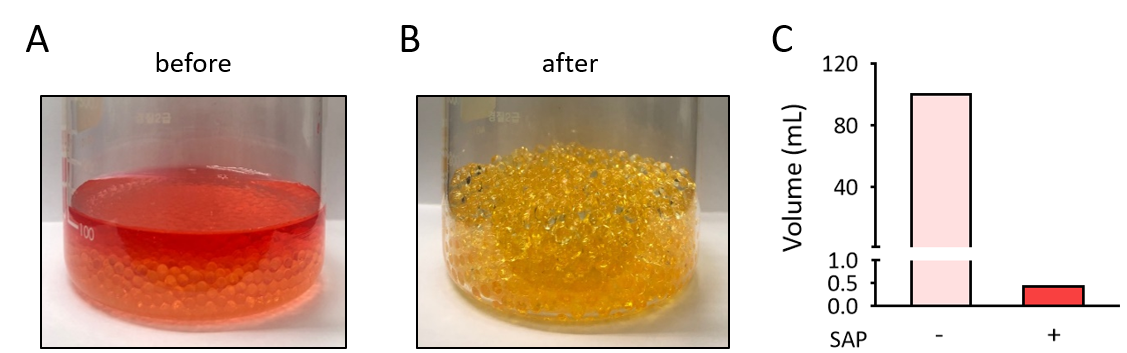


**Fig. S10. Concentration of 100 mL culture medium by SAP beads** (A, B) 100 mL cell culture medium was tested for large volume reduction by SAP beads. 250 mg/mL SAP beads were incubated with culture medium for 0 min (A) and 30 min (B). (C) The % volume reduction was calculated. Note that most of the culture medium was absorbed to the beads.
